# Invasion of HEp-2 cells by Shigella spp. isolated from acute pediatric diarrhea

**DOI:** 10.3205/id000031

**Published:** 2017-09-15

**Authors:** Sajjad Omidi, Mohammad Mehdi Soltan Dallal, Abolfazle Davoodabadi, Ramin Mazaheri Nezhad Fard, Marayam Usefi, Ronak Bakhtiari

**Affiliations:** 1 Division of Microbiology, Department of Pathobiology, School of Public Health, Tehran University of Medical Sciences, Tehran, Iran; 2 Division of Food Microbiology, Department of Pathobiology, School of Public Health, Tehran University of Medical Sciences, Tehran, Iran; 3 Food Microbiology Research Center, Tehran University of Medical Sciences, Tehran, Iran; 4 Department of Microbiology, Medical School, Babol University of Medical Science, Babol, Iran; 5 Department of Virology, School of Public Health, Tehran University of Medical Sciences, Tehran, Iran

**Keywords:** cell invasion, cell culture, diarrhea, Shigella spp., HEp-2

## Abstract

**Aim:** *Shigella* infection is an important global health problem in developing countries where hygiene is poor and hence shigellosis is a main cause of diarrhoea-associated mortality and morbidity, particularly in children under the age of five. The bacterial entry into colon and rectal epithelial cells has been named ‘bacterium-directed phagocytosis’. This term highlights that the bacteria actively stimulate their own uptake into non-professional phagocytes. The aim of this study was to demonstrate the invasion of HEp-2 cells by *Shigella* spp. isolated from acute pediatric diarrhea in Tehran, Iran.

**Methods:** Three-hundred and ten non-duplicative diarrheal stool samples were collected from the children admitted to Children’s Medical Center in Tehran, Iran. Samples were cultured and suspected colonies were identified by routine microbiological and biochemical tests. The invasion of the two isolated *Shigella* spp. to HEp-2 cells was studied.

**Results:** Of 310 stool samples, 16 (5.2%) *Shigella* spp. were isolated, including seven (43.7%) *S. sonnei* and nine (56.3%) *S. flexneri*. Four (44.4%) *S. sonnei* and seven (42.8%) *S. flexneri* showed invasive phenotype to HEp-2.

**Conclusion:** *Shigella sonnei* and *S. flexneri* are reported as the most prevalent *Shigella* spp. in nature which infect humans. Invasion of various cell lines gives the chance of survival to *Shigella* spp. This ability causes more virulent infections in the host. Despite costly and time consuming cell culture techniques, the current method described in this paper is reliable for detecting invasive behavior of *Shigella* spp. Results have also shown that not all the *Shigella* spp. are able to invade intestinal epithelial cells.

## Introduction

*Shigella* infection or shigellosis is a significant global health problem in developing countries where hygiene is poor. Shigellosis is a main cause of diarrhoea-associated mortality and morbidity, particularly in under five-year-old children [1]. *Shigella* spp. are categorized in four serogroups, including *S. dysenteriae* (serogroup A), *S. flexneri* (serogroup B), *S. boydii* (serogroup C) and *S. sonnei* (serogroup D). The bacteria are responsible for causing gastroenteritis in the host that may progress to mucoid bloody diarrhea, known as bacillary dysentery [2]. *Shigella flexneri* and *S. sonnei* have been described as the most common causes of shigellosis in tropical areas. Furthermore, *S. sonnei* is mainly isolated in developed countries. Recently, it has been estimated that 91 million cases of *Shigella* infections occur every year. In Asia, 410,000 children, commonly undernourished, die every year due to *Shigella* infections. *Shigella* spp. are transmitted through the fecal-oral route and enter the human body via the ingestion of contaminated food and water [3]. *Shigella* spp. cause bacillary dysentery in humans by invading epithelial cells of the colon. The bacterial entry into colon and rectal epithelial cells has been named ‘bacterium-directed phagocytosis’. This term describes that the bacteria actively stimulate non-professional phagocytes to engulf them. Bacterial invasion proteins IpaB, IpaC and IpaD are necessary for the process [4]. *Shigella* spp. have a large (100–140 MDa) plasmid, which is critical for their virulence and at least three chromosomal loci are required for the bacterial pathogenesis. The intracellular entry of *Shigella* spp. into the host cells is not passive and needs the expenditure of energy by the bacteria and their host cells. Following entry, *Shigella* quickly lyses phagosomal membrane and replicates in the host cell cytoplasm [5]. Bacteria move efficiently in infected cell cytoplasm by polymerization of actin at bacterial pole, which also allows formation of protrusions in cell membrane leading to invasion of adjacent cells [6]. The aim of the current study was to demonstrate invasion of HEp-2 cells by *Shigella* spp. isolated from acute pediatric diarrhea in Tehran, Iran.

## Methods

### Clinical samples and bacterial isolation

Three-hundred and ten non-duplicative diarrheal stool samples were collected from January to December 2015 from 0- to 12-year-old children (165 males and 145 females) admitted to Children’s Medical Center in Tehran, Iran. Suspected colonies were identified by routine microbiological and biochemical tests, including API-20E system kit (BioMerieux, France) and Shigella polyvalent agglutinating antisera (MAST, UK).

### Cell culture adherence and penetration 

HEp-2 cells were chosen because of their extensive use and accessibility. *Shigella* isolates were cultured in brain heart infusion (BHI) agar. HEp-2 cells were preserved in Dulbecco modified Eagle medium (DMEM) with 10% fetal calf serum (FCS). Confluent monolayers of 5.0×10^4^–10^5^ HEp-2 cells per ml were grown for 18 h in 6-well tissue plates at 37°C in humidified incubator containing 5% CO_2_. To infect HEp-2 cells, bacterial isolates were suspended in DMEM with 10% FCS without antibiotics to give a final concentration of approximately 5×10^5^ cells per ml. One milliliter of this suspension was added to the monolayers. The infected monolayers were incubated for 2 h and then washed three times with 2 ml of phosphate buffered saline (PBS). For intracellular growth step, fresh DMEM containing 100 µg/ml of gentamicin was added to the monolayers and incubated for further 3 h and then washed three times with PBS. Cell monolayers were washed in PBS, fixed in a mixture of 3:1 methanol/acetic acid for 10 min and stained with Giemsa, then examined under an invert microscope. Invasion index (penetration to the cells) was recorded as 10–30 (1^+^), 30–70 (2^+^) and 70–100 (3^+^). *S. flexneri* ATCC 12022 and *S. sonnei* ATCC 9290 were used as positive and *Escherichia coli* K1 as negative control.

## Results

### Clinical samples and bacterial isolation

Of 310 stool samples from children with diarrhea, 16 (5.2%) *Shigella* spp. were isolated. Ten isolates were identified in male and six in female children. Slide agglutination test using monovalent antisera showed that seven (43.7%) *S. sonnei* and nine (56.3%) *S. flexneri* were identified out of the 16 positive samples. The mean age of the patients was six years with 165 (53.2%) male and 145 (46.7%) female participants. Nine (56.2%) strains were isolated from children in ages ranged from one month to two years and seven (43.7%) from those in ages from two to 12 years.

### Cell culture adherence and penetration

**Figure 1:**
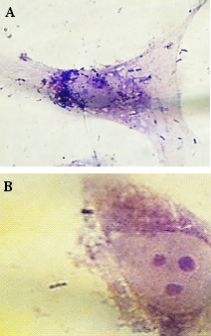
Gentamicin-HEp-2 cell invasion assay. Intracellular localization of *S. sonnei* (A) and *S. flexneri* (B) in HEp-2 cell monolayers after 2 hours

**Figure 2:**
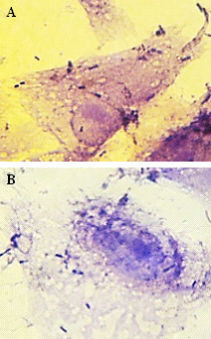
Gentamicin-HEp-2 cell invasion assay. Intracellular localization of *S. sonnei* (A) and *S. flexneri* (B) in HEp-2 cell monolayers after 3 hours

Four (44.4%) isolates of *S. sonnei* and seven (42.8%) isolates of *S. flexneri* showed invasive phenotype to HEp-2. Ability of *Shigella* isolates to invade HEp-2 cell monolayers was first assessed using microscopic examination of the monolayer and detection of Giemsa-stained intracellular bacteria. Invasion index (penetration in the cells) was recorded as 10–30 (1^+^), 30–70 (2^+^) and 70–100 (3^+^). After 2 h of incubation, *Shigella* isolates could invade cells, but replication in and destruction of the cells occurred after 3 h of incubation (Figure 1 (Fig. 1) and Figure 2 (Fig. 2)).

## Discussion

Approximately 91 million people are infected by *Shigella* spp. worldwide each year [7]. It is well established that *S. flexneri* is the predominant bacterial isolate in developing countries; in contrast, *S. sonnei* is the most common bacterial isolate in developed countries [8]. There has been dramatic change in predominant bacterial strain in some Asian and African countries such as Bahrain, Iran, Thailand and Vietnam, in which *S. flexneri* predominance has shifted to *S. sonnei* predominance [9], [10], [11], [12], [13]. In the current study, 16 (5.2%) *Shigella* spp. were isolated from 310 non-duplicative stool samples, from which seven (43.7%) isolates included *S. sonnei* and nine (56.3%) isolates included *S. flexneri*. These results differ from the results by Eftekhari et al. [14]. In a study by Eftekhari et al., a total number of 32 (4.5%) *Shigella* spp. were recognized in 700 stool samples from patients with diarrhea in two provinces in Iran. *S. sonnei* (70.8%) and *S. flexneri* (62.5%) were the most prevalent species in Tehran and Khorasan Razavi Provinces, respectively [14]. However, the difference between the results of the two studies might be seen due to the sample size. However, the prevalence rates of *Shigella* spp. in both studies are relatively similar (5.2% compared to 4.5%).

Invasion of gastrointestinal epithelia is one of the major virulence mechanisms, by which Gram-negative bacteria cause diarrheal diseases [15]. A majority of enteric pathogens, including *Shigella* and *Salmonella* spp., have been shown to possess such mechanisms. These mechanisms can be reproduced *in vitro* by indicating the ability of virulent strains to invade mammalian cell lines such as HEp-2 or HeLa [16]. Although CHO cell line has been used to study the bacterial invasion, this cell line is sensitive to the effects of bacterial toxins. The susceptibility of CHO cells to toxins has made these cells inappropriate for the investigation of elongated bacterial infections [17]. Another cell line, Madin-Darby canine kidney (MDCK), seems more appropriate for the study, but is less susceptible to bacterial invasion [18]. A comparative study has shown that Henle 407 cell line is highly efficient in adherence and invasion of *S. flexneri*. Although HeLa cells are used to investigate invasion of *Shigella* spp., Henle 407 cells have been shown to be more efficient [19]. Research has shown that *Shigella* spp. degrade cell cultures in replication phase. Furthermore, attachment and invasion of *Shigella* spp. is inhibited by low concentration of IgA [20], [21], [22]. In the current study, Hep-2 cells were used to demonstrate the invasion of *Shigella* spp. Of 16 *Shigella* spp., four (44.4%) *S. sonnei* and seven (42.8%) *S. flexneri* isolates showed invasive phenotype to HEp-2 after 2 h of incubation. However, replication in and destruction of the cells occurred completely after 3 h. These results are relatively similar to those of a study by Soltan Dallal et al. in 2013 [23]. They isolated 36 (8.6%) *Shigella* spp. from 280 rectal swabs (140 dysentery and 140 watery diarrheal samples) and reported that 14 (38.8%) isolates demonstrated invasive phenotype to HEp-2. Previous studies have shown that it is essential to primarily grow the bacteria at 37°C for the optimal expression of invasion phenotype of *S. flexneri* on the HEp-2 cell line. Results from the current study have revealed the invasive ability of *Shigella* spp. and demonstrated that a 5-h incubation time is sufficient for complete destruction of the cell layers by *Shigella* spp. 

## Conclusions

Despite costly and time consuming cell culture techniques, the current described technique is a reliable technique for detecting invasive behavior of the *Shigella* spp. This study actually provides a good model for further studies on the invasive properties of these bacteria. In conclusion, although cell invasion is described as a common feature of the highlighted *Shigella* species as detected in a majority of the isolates within the current study, this feature was not detected in a minor portion of the total bacterial samples.

## Notes

### Acknowledgments

This work was supported by a Vice-Chancellor for Research grant (No. 23125), Tehran University of Medical Science, Tehran, Iran. We thank Children’s Medical Center in Tehran for providing bacterial isolates and related data for use in this study. 

### Competing interests

The authors declare that they have no competing interests.
